# Supplementary material for: The molecular properties of the bHLH TCF4 protein as an intrinsically disordered hub transcription factor
Source: Cell Commun Signal. 2025 Mar 27;23:154. doi: 10.1186/s12964-025-02154-7 (PMC11948756; doi:10.1186/s12964-025-02154-7)
Supplement: Supplementary file 1 — Supplementary Material 1 [file 12964_2025_2154_MOESM1_ESM.docx]

**
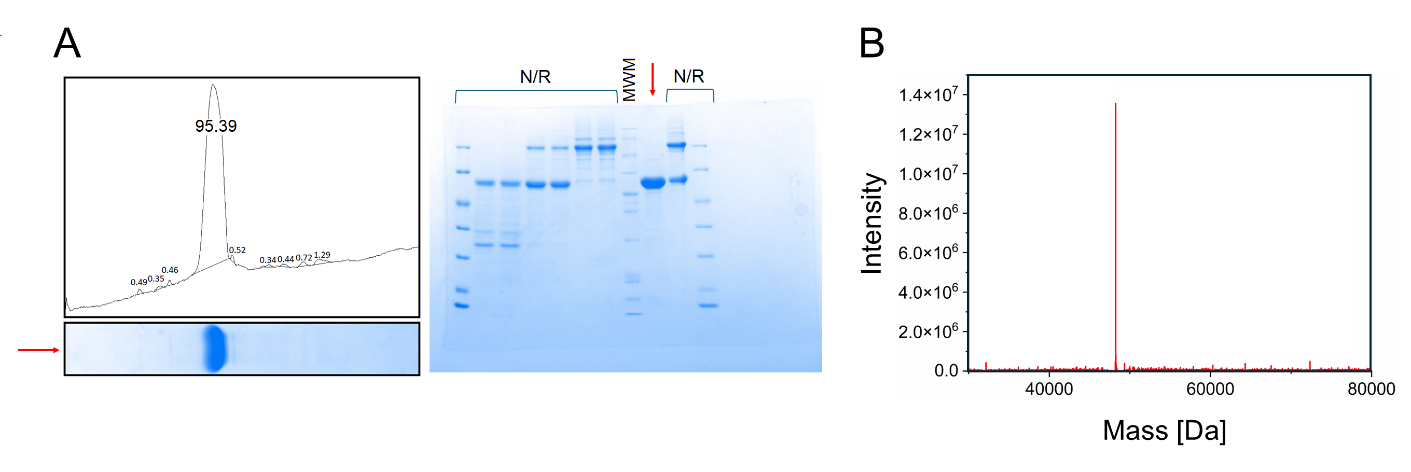
**

**Fig. S1. Determination of the purity of the TCF4 sample.** (A) Estimation of the purity of the TCF4 sample (red arrow) using ImageJ (left) from SDS-PAGE gel stained with Coomassie Brilliant Blue R-250 (right). The estimated purity of TCF4 was approximately 95%. N/R = not relevant; data that do not contribute to the analysis, because they are unrelated to the context. MWM – Spectra™ Multicolor Broad Range Protein Ladder (Thermo Scientific) (B) TCF4 mass determination via ESI MS. The obtained value equals the theoretical mass of recombinant TCF4 I^-^ (UniProt ID P15884-16) with an additional N-terminal Met.

| 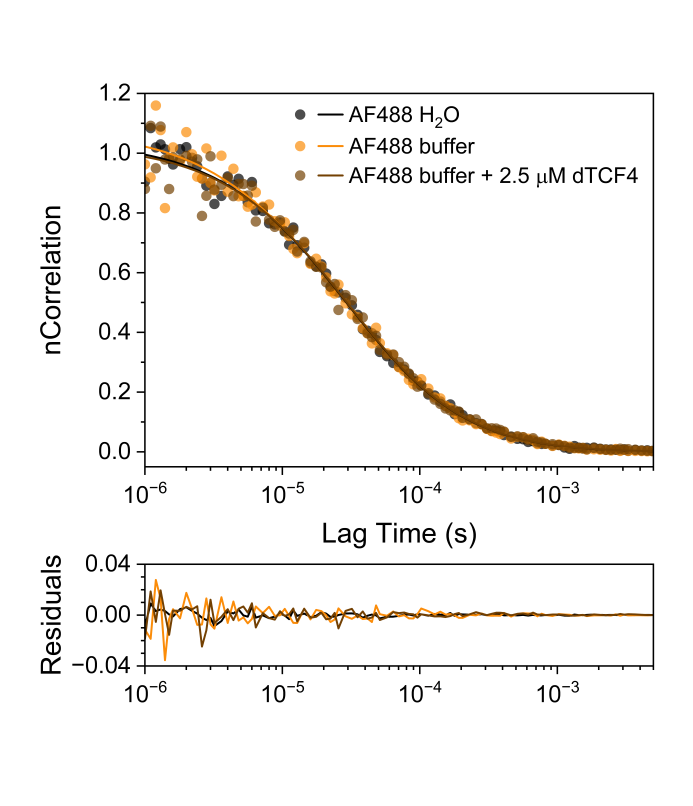 | **Fig. S2. Control FCS measurements** of the diffusion times of the AF488 fluorescent probe in different solutions to account for the increase in viscosity due to the presence of the dTCF4 protein. |
| --- | --- |

**Table S1. Diffusion times of the AF488 fluorescent probe in different solutions.** The presence of the highest concentration of dTCF4 led to an increase of solution viscosity of about 6%. The FCS data shown in **Figure 3** have been corrected accordingly.

|  | **tau AF488 [us]** | | | **tau AF488 with prot / in buf** |
| --- | --- | --- | --- | --- |
|  | **ddH2O** | **buffer** | **buffer with 2.5 uM dTCF4** |  |
|  | 30.2269 | 27.6073 | 29.8726 |  |
|  | 28.5574 | 28.1975 | 29.8995 |  |
|  | 29.9757 | 29.2268 | 30.2651 |  |
| **Mean** | **29.6** | **28.3** | **30.0** | **1.059** |
| SD | 0.9 | 0.8 | 0.2 | 0.032 |
| SEM | 0.5 | 0.5 | 0.13 | 0.018 |

**
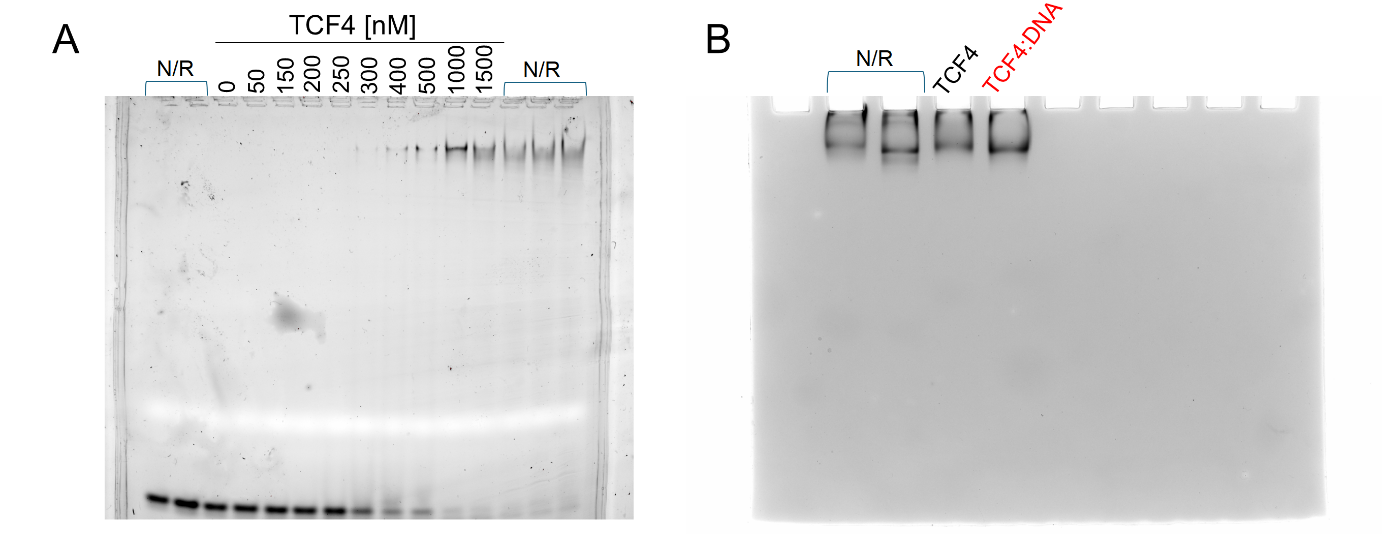
**

**Fig. S3. TCF4 native PAGE.** (A) E-box binding properties of TCF4 analyzed by EMSA. 10 μl of samples containing a FAM-labeled dsDNA probe (40 nM), TCF4 (0–1.5 µM per dimer), glycerol (final concentration of 20%) and bromophenol blue (final concentration of 1%) were loaded onto 5% native polyacrylamide gels and run at 150 V for 150 min in 0.5 × TBE buffer at 4 °C. Images were captured using the ChemiDoc MP Imaging System (Bio-Rad). (B) Results of TCF4 electrophoresis using 5% native polyacrylamide gel. 20 μl of samples of free TCF4 (2 μM) and TCF4 (2 μM) with E-box (20 μM) were mixed with glycerol (20%) and bromophenol blue (1%). The samples were then loaded onto a gel, and run at 150 V for 40 min in 0.5 × TBE buffer at 4 °C. After electrophoresis, the gel was stained with Coomassie Brilliant Blue R-250 and analyzed using Image Lab Software (Bio-Rad). N/R = not relevant; data that do not contribute to the analysis, because they are unrelated to the context.


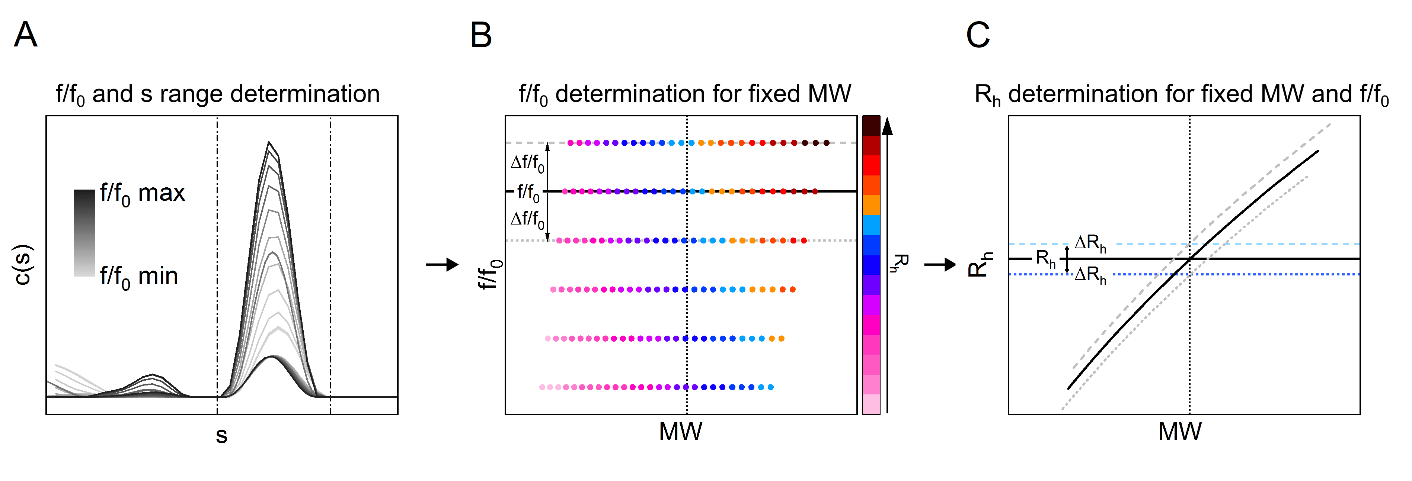


**Fig. S4. Method of determination of R_h_ from SV-AUC** using the continuous c(f/f_0_) distribution for two or more entities in the sample based on their known molecular weight (MW).


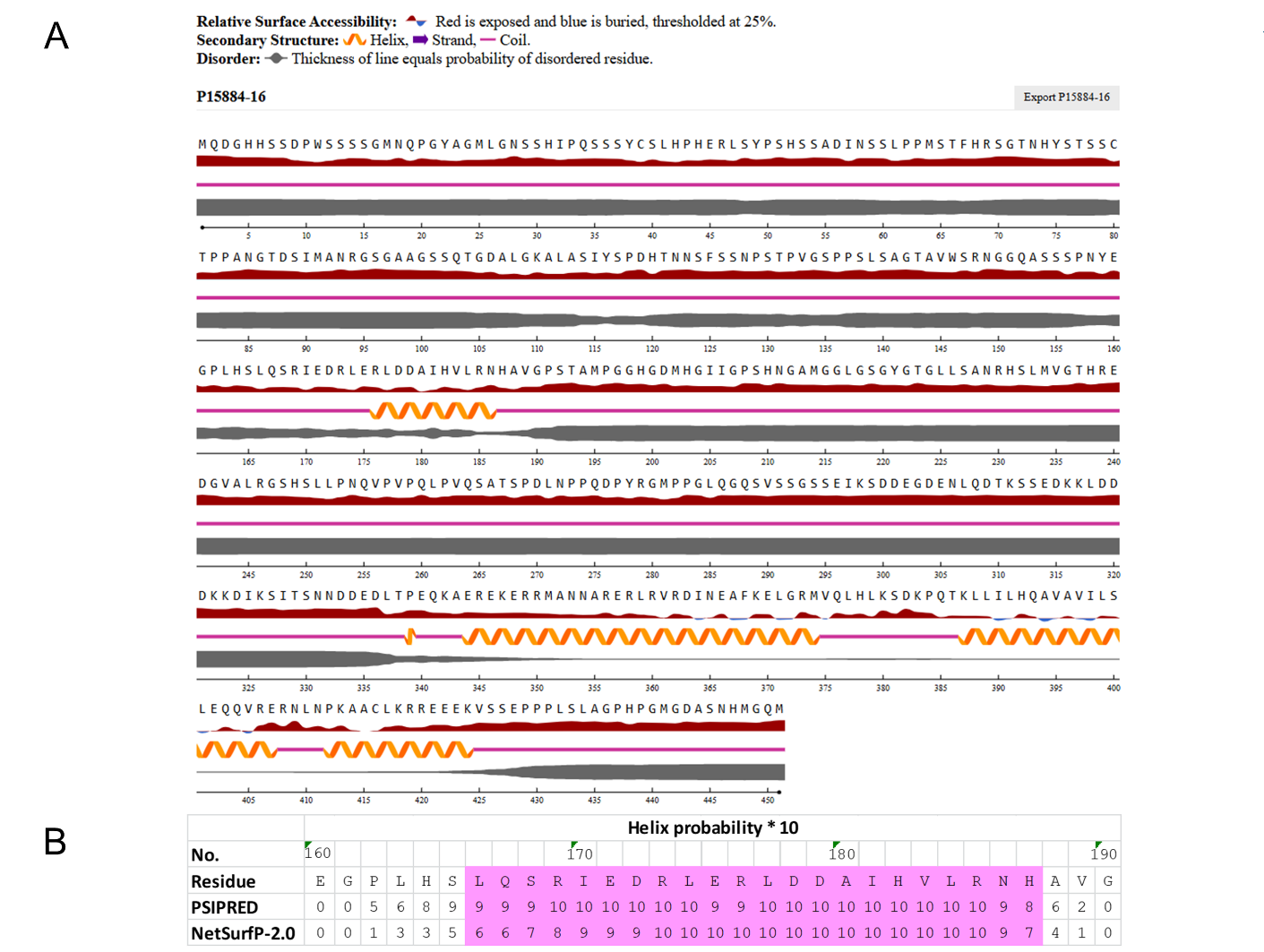


**Fig. S5. TCF4 NetSurfP-2.0 and PSIPRED prediction result.** (A) According to the NetSurfP-2.0 results for TCF4, all the residues involved in the formation of secondary structures form the helices. (B) α-helix probability detected for the 166–187 fragment of TCF4 by PSIPRED and NetSurfP-2.0. The consensus predictions are marked in pink.

| 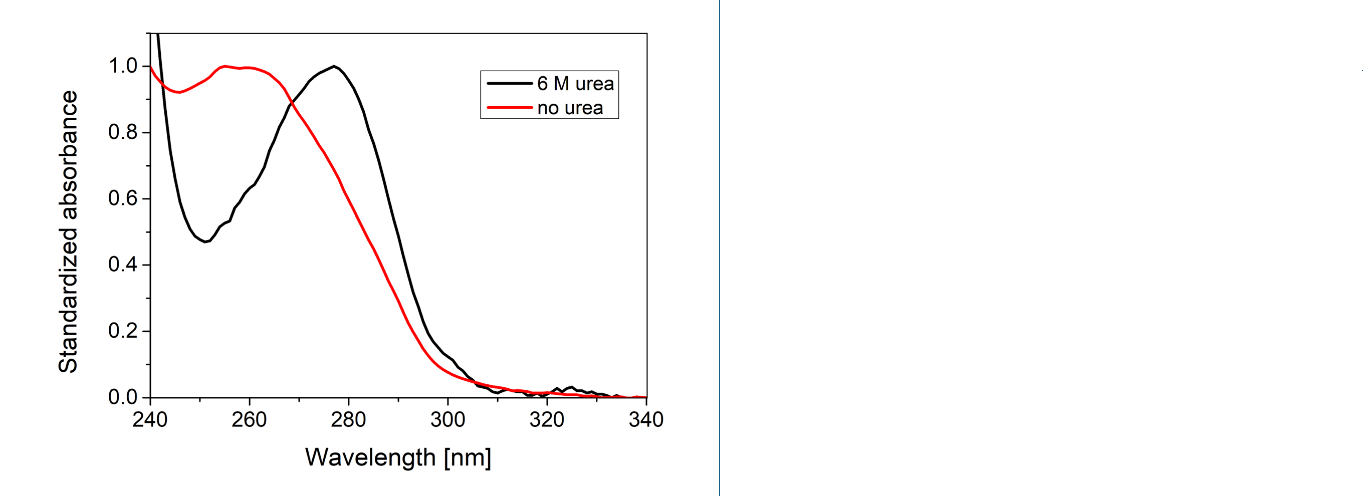 | **Fig. S6. UV spectra of TCF4 purified using the 6 M urea rinse step (black) and omitting this step (red).** The apparent shift is due to the presence of nucleic acids in the sample that was not denatured. |
| --- | --- |

| 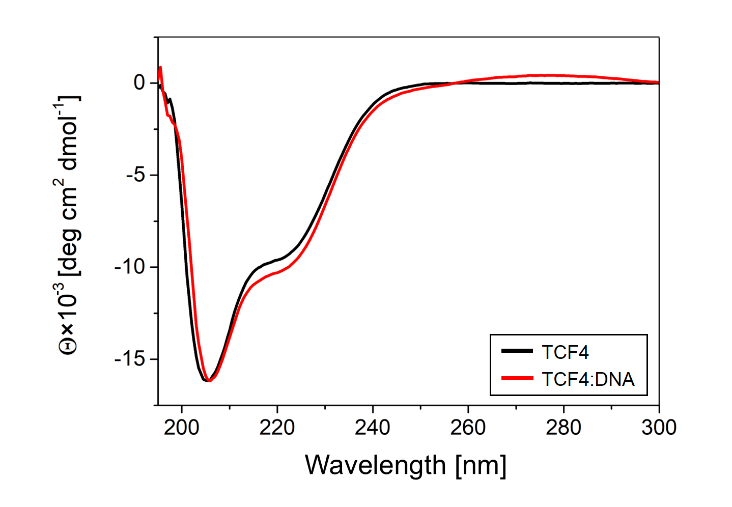 | **Fig. S7. CD spectra of TCF4.** TCF4 in both DNA-bound and free form shows a deep minimum around 205 nm and a shoulder at 222 nm typical for a random coil conformation with some contribution of an α-helical structure. In the case of DNA-bound TCF4, the shoulder around 222 nm becomes deeper and the minimum is slightly shifted to 208 nm, suggesting a disorder-to-order transition towards the α-helical fold. A weak signal is also observed at about 280 nm, coming from DNA molecules present in the sample. |
| --- | --- |


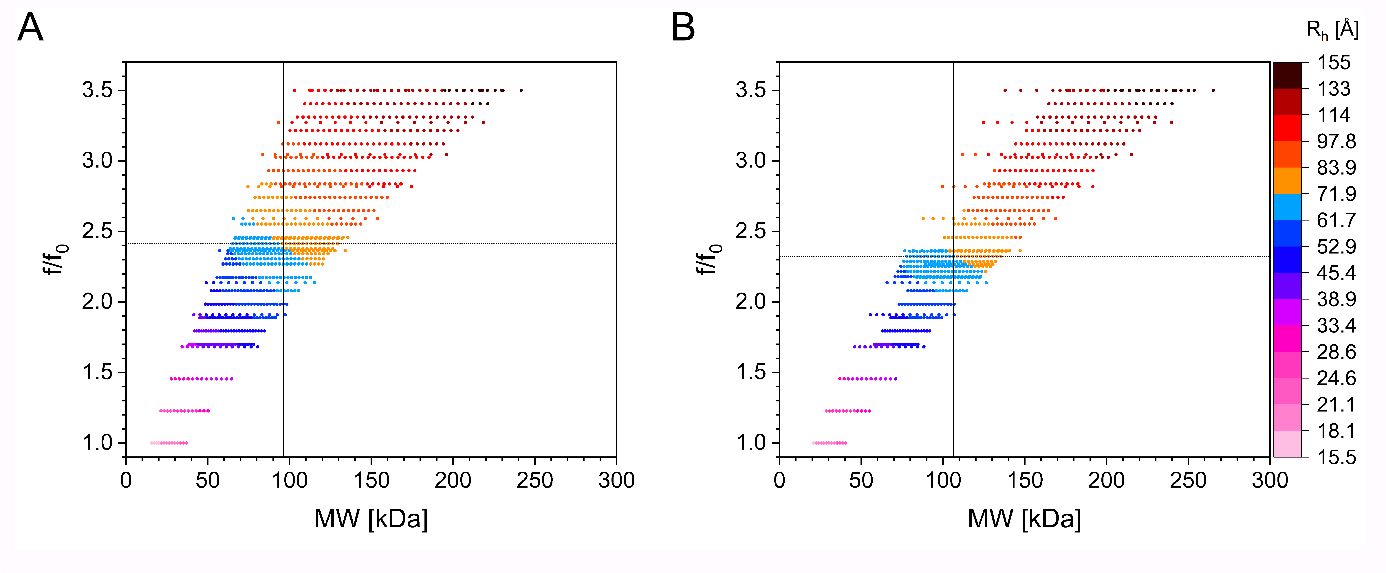


**Fig. S8. Determination of the f/f_0_ shape factor value, for which the molecular weight distribution generated by SEDFIT centers on the known MW values of (A) dTCF4 or the (B) dTCF4:dsDNA complex.**

**
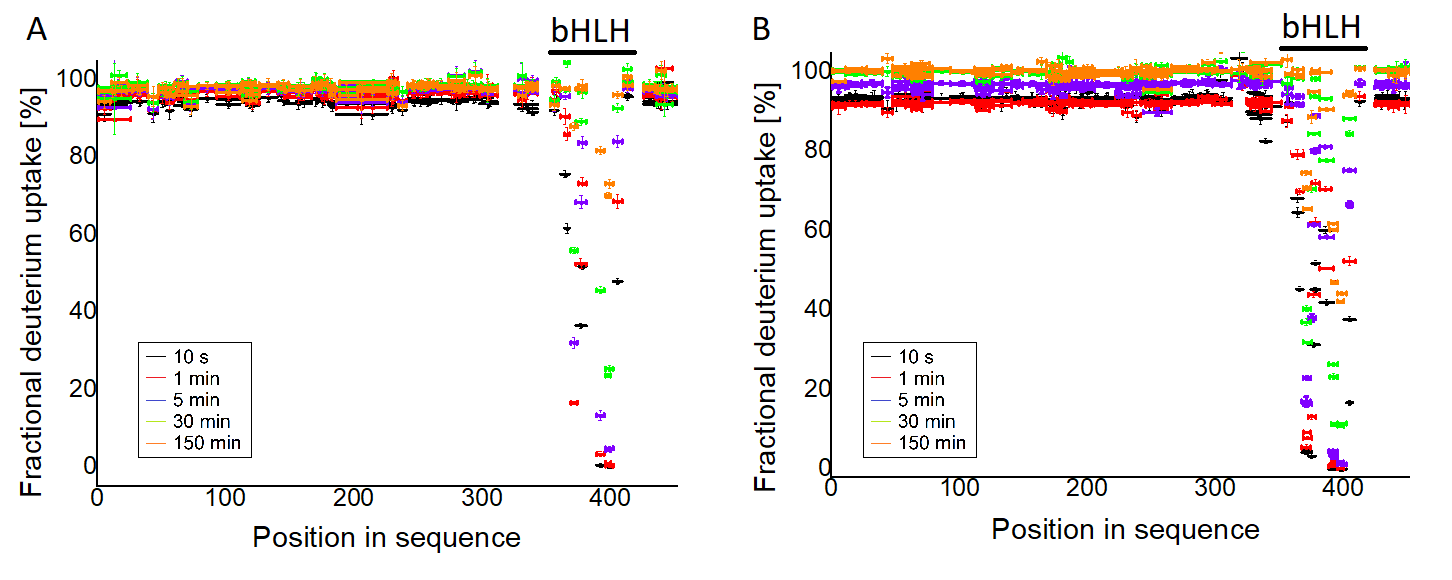
**

**Fig. S9. HDX analysis of TCF4** (A) purified with the use of denaturing agent; (B) purified without denaturing agent. In the latter case, the protein was significantly contaminated with bacterial nucleic acids.
