# Supplementary figures and images for: The molecular properties of the bHLH TCF4 protein as an intrinsically disordered hub transcription factor
Source: Cell Commun Signal. 2025 Mar 27;23:154. doi: 10.1186/s12964-025-02154-7 (PMC11948756; doi:10.1186/s12964-025-02154-7)

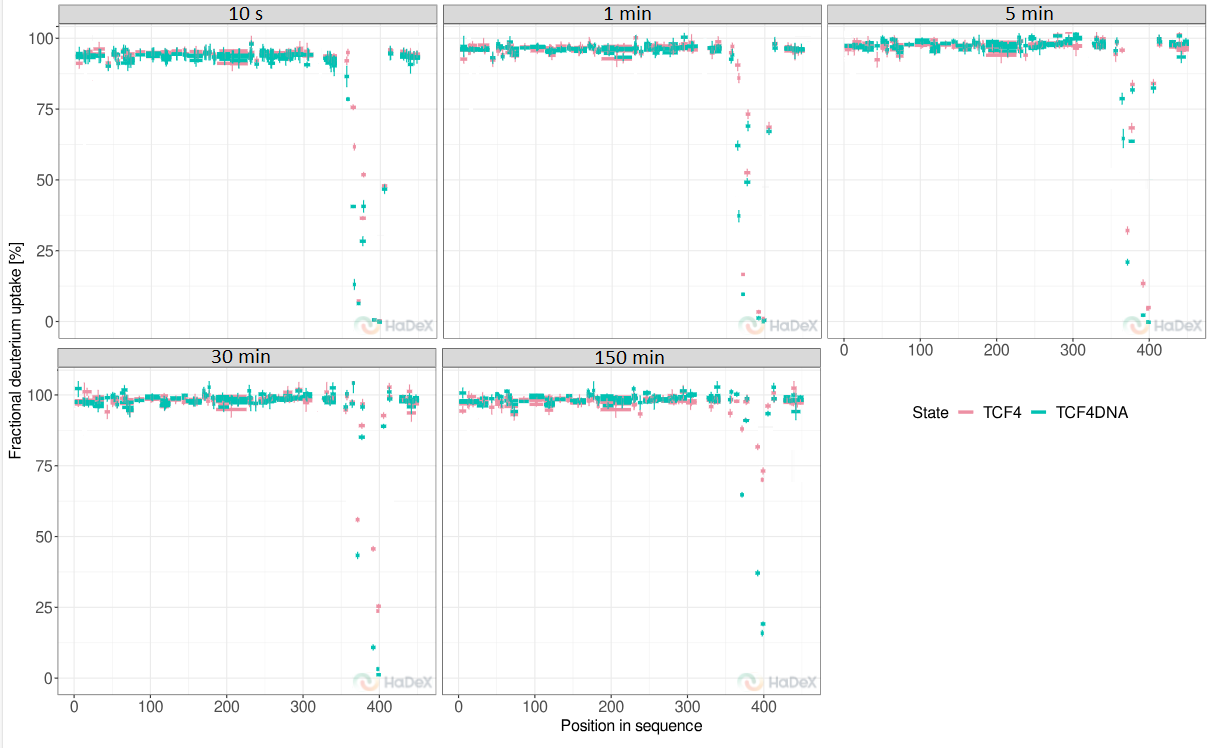

Supplement: Supplementary file 2 — Supplementary Material 2 [file 12964_2025_2154_MOESM2_ESM.tif]
